# Evaluation of the Learning Curve in Robotic-Assisted Total Knee Arthroplasty: A Time-Series Analysis of Surgical Time

**DOI:** 10.7759/cureus.84120

**Published:** 2025-05-14

**Authors:** Maximiliano Barahona, Felipe Bustos, Jaime Hinzpeter, Francisco Urroz, Cristian Barrientos, Carlos A Infante, Macarena A Barahona

**Affiliations:** 1 Department of Orthopaedics, Hospital Clínico Universidad de Chile, Santiago, CHL; 2 Department of Orthopaedics and Traumatology, Hospital Clínico Universidad de Chile, Santiago, CHL

**Keywords:** learning curve, robotic assistance, rosa, surgical time, time series analysis, total knee arthroplasty

## Abstract

Objective

This study aimed to assess whether surgical times in robotic-assisted total knee arthroplasty (rTKA) could be comparable to those of conventional total knee arthroplasty (TKA), and to identify the point in the learning curve at which this occurs.

Methods

A time-series analysis was conducted on the first 50 consecutive primary rTKA procedures performed by a single surgeon at a university hospital. The surgeon, with seven years of experience and performing 30 TKA surgeries annually, used the ROSA^®^ (Zimmer Biomet, Warsaw, IN, USA) robotic system for all procedures. Surgical times were analyzed using a Markov switching model to detect significant changes in operative duration. A Kruskal-Wallis test was applied to compare surgical times between rTKA and conventional TKA groups, with post hoc analysis conducted to assess differences between the first and second epochs of rTKA and conventional TKA.

Results

Markov analysis revealed two distinct epochs in surgical performance, with a significant reduction in surgical time occurring after the 20th case. The median surgical time in the first epoch was 118 minutes, compared to 105 minutes in the second epoch. The conventional TKA group had a median surgical time of 100 minutes. Statistical analysis, using the Kruskal-Wallis test, identified significant differences among the groups, with post hoc testing revealing no significant difference between the second epoch of rTKA and conventional TKA.

Conclusions

Surgical times for rTKA become comparable to those of conventional surgery after approximately 20 cases. These findings suggest that rTKA does not negatively impact operating room efficiency once the surgeon gains proficiency with the technology.

## Introduction

Total knee arthroplasty (TKA) is a common orthopedic surgery for advanced osteoarthritis [1]. As the global population ages, the demand for TKA is expected to reach 3.5 million procedures per year by 2030 [2]. Despite a growing satisfaction rate, which now approaches 90% [3] and is comparable to that of total hip arthroplasty [4], a subset of patients continues to report dissatisfaction with the outcome.

To stay at the cutting edge, the use of robotic technology in TKA has gained attention for its ability to improve surgical precision, enable detailed preoperative planning, and provide real-time intraoperative feedback [5-7]. Although robotic-assisted systems have advanced technologically and become more widely available, their adoption in TKA has not been universal [8]. The learning process for new technologies generates stress for both surgeons and surgical staff, which increases operative times and the risk of complications [9].

One concern is the perception that robotic assistance may extend operative times, potentially increasing the risk of complications such as infections or thromboembolism, and reducing operating room efficiency [10,11]. However, studies such as those by Naziri et al. [12] have shown that, for surgeons without prior robotic experience, robotic assistance does not increase surgical risks and achieves operative times comparable to conventional techniques after the initial 20 cases. Similarly, Vanlommel et al. [13] reported that the learning curve for operative times ranges between 6 and 11 cases, with no significant differences in complication rates.

While these studies provide valuable insights into the adoption of robotic-assisted surgery, it is important to note that they rely on traditional mean comparison methods, such as t-tests, which require data independence [14]. In the context of surgical learning curves - where operative times for a surgeon are inherently dependent on prior cases due to the cumulative learning process - a time-series analysis may offer a more appropriate framework. This approach accounts for the sequential nature of the data and provides a more nuanced understanding of how surgical performance evolves over time [15].

A key challenge in implementing robotic assistance in TKA (rTKA) is the potential for increased surgical duration (Tcx). To address this, the present study has three main objectives: (1) to identify the number of cases required for a significant reduction in surgical time during the adoption of rTKA, using time-series analysis; (2) to determine whether the surgical time achieved after this point of significant reduction is comparable to that of conventional TKA. Together, these analyses allow us to quantify the learning curve and evaluate whether surgical efficiency with robotic assistance can ultimately match that of conventional techniques; (3) to evaluate the safety and alignment outcomes of rTKA by assessing postoperative complications, 60-day hospital readmissions, and the hip-knee-ankle (HKA) angle. These metrics ensure that the improved efficiency observed with rTKA does not come at the cost of alignment accuracy or patient safety.

## Materials and methods

This research is a time-series analysis of the initial 50 consecutive cases of primary rTKA conducted by a single surgeon at a university hospital. The surgeon has seven years of experience and performs approximately 30 standard primary TKA procedures annually, in addition to around 10 unicompartmental knee arthroplasties and five revision or complex primary cases. Prior to initiating clinical use of the robotic system, the surgeon completed a dedicated two-day training course that included both theoretical instruction and hands-on simulation, as well as expert-led discussions on rTKA. The study received approval from the institution’s ethics committee (approval no. 40/2021).

The inclusion criteria were primary rTKA using ROSA^®^ (Zimmer Biomet, Warsaw, IN, USA), regardless of whether a posterior-stabilized (PS) or cruciate-retaining (CR) implant was used. Patients were excluded if a more constrained prosthesis design was required or if there was a history of previous osteosynthesis around the knee. Additionally, patients with incomplete records of surgical time were excluded. Preoperative coronal deformity was not considered a selection criterion. Patients were identified through a prospective registry, and their medical records and imaging were reviewed.

**Figure 1:**
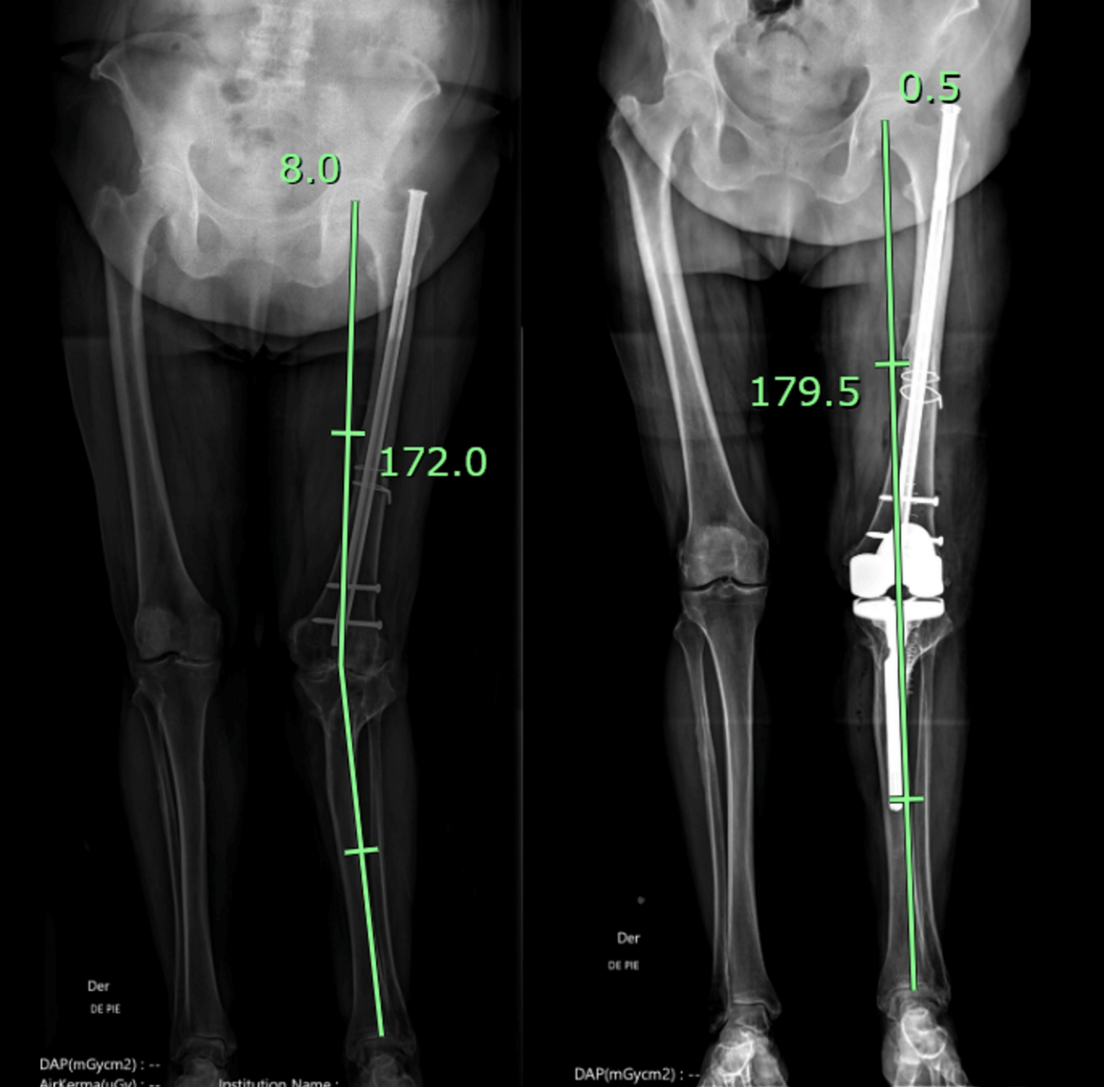
Example of a complex primary TKA excluded from the learning curve analysis Preoperative (left) and postoperative (right) standing long-leg radiographs of a patient with a complex primary TKA were excluded from the analysis. This case involved a prior distal femoral fracture treated with osteosynthesis, resulting in a significant extra-articular deformity. The robotic system was used strategically to plan femoral cuts that avoided nail removal, allowing safe implant positioning without hardware extraction. On the tibial side, the robot was utilized to perform a precise resection at 90° in the coronal plane and 0° of posterior slope, creating a flat surface to facilitate subsequent preparation and implantation of a stemmed tibial component using conventional instruments. Postoperative alignment was successfully restored (HKA: 0.5°). Given the deviation from the standard workflow of primary TKA and the additional complexity involved, this case was classified as a complex primary and excluded from the learning curve analysis, which focused exclusively on standard primary rTKA procedures. TKA, total knee arthroplasty; HKA, hip-knee-ankle; rTKA, robotic-assisted total knee arthroplasty

Three patients did not meet the inclusion criteria: one patient had a femoral stem and sequelae of a tibial fracture requiring a tibial stem, while the other two cases required a more constrained insert and stem due to severe valgus (Figure 1).

Thus, the series includes 50 consecutive cases of primary rTKA but encompasses up to the 53rd case performed by the surgeon using robotic assistance. No patient was excluded for missing data in their medical record. 

Functional alignment was used in performing rTKA, with limits set at 5° of varus and valgus, via a medial parapatellar approach. No tourniquet or patellar resurfacing was used, consistent with the surgeon’s preference. All patients received standardized preoperative management, including 2 g of intravenous cefazolin and 1 g of tranexamic acid. Spinal anesthesia was administered in all cases, complemented by an adductor canal block and an iPACK (Infiltration between the Popliteal Artery and Capsule of the Knee) block for postoperative analgesia.

The procedure began with a standard medial parapatellar arthrotomy, followed by selective soft tissue release to allow adequate patellar eversion. Only the necessary portion of Hoffa’s fat pad was excised to expose the anterior cruciate ligament and the anterior horn of the lateral meniscus. The anterior portion of both menisci was resected, and the posterior cruciate ligament was preserved. Once the patella was everted, pins for robotic referencing were placed within the surgical incision, and the ROSA^®^ system was initialized. Anatomical landmarks were then registered on the femur and tibia as required by the system.

The patient’s mechanical axis and soft tissue balance were assessed in full extension and at 90° of flexion, including varus-valgus laxity in both positions. If significant discrepancies were noted with the surgeon’s intraoperative assessment or the preoperative radiographs, anatomical landmarks were re-registered. Component positioning was subsequently adjusted - modifying femoral and tibial alignment (varus/valgus), slope, femoral component size, and femoral rotation - to balance medial and lateral gaps in both flexion and extension, targeting symmetric gaps of 18.5 mm, with a maximum tolerated difference of 1.5 mm.

If optimal balance was achieved during the planning phase, bone resections were performed as follows: all femoral cuts first, followed by the live tibial resection (without the use of fixation pins). If the initial robotic plan did not result in balanced gaps, the extension gap was first set to 18.5 mm, and both the distal femoral and tibial resections were performed as live cuts. The flexion gap was then reassessed and rebalanced using the robotic platform before completing the remaining femoral resections.

Following surgery, patients were allowed to begin active range-of-motion exercises as soon as spinal anesthesia had worn off and were mobilized to stand and walk upon arrival to their hospital room, typically around four hours postoperatively. During hospitalization, patients underwent an average of four physical therapy sessions and were discharged approximately 48 hours after surgery.

The primary outcome was the evolution of surgical time. Surgical time was recorded in the electronic medical record by the operating room nurse, beginning when the surgeon started the incision and ending when the dressings were applied. This recording method has been in place since the introduction of the electronic medical record system in 2012.

All patients underwent postoperative evaluation with standing full-length anteroposterior radiographs of the lower limb (long-leg radiographs) to measure the HKA angle. This angle was recorded as negative in cases of valgus alignment and positive in cases of varus alignment. Furthermore, each patient's medical chart was reviewed to identify postoperative complications, including infection, wound dehiscence, thromboembolic events, or hospital readmissions within 60 days after surgery. These data were included to ensure that improvements in surgical efficiency throughout the robotic learning curve were not associated with compromised alignment accuracy or an increased rate of early complications.

To address the primary objective of this study - evaluating the evolution of surgical time during the adoption of rTKA - a longitudinal time-series analysis was conducted. A Markov-switching model was used to examine transitions in surgical performance across consecutive cases, allowing the identification of distinct phases (or "epochs") based on changes in surgical time patterns [16]. To statistically determine the inflection point at which surgical time significantly decreased - corresponding to the second objective - we applied the Supremum Wald (Sup-Wald) test, a method specifically designed to detect structural breaks in longitudinal data.

To address the third objective, surgical times from a comparison cohort of 75 consecutive standard primary TKAs performed by the same surgeon using conventional instrumentation were analyzed. The same inclusion and exclusion criteria applied to the robotic group were used: revision procedures, complex primary cases (e.g., severe deformities or retained hardware), and unicompartmental knee arthroplasties were excluded. These conventional TKAs were performed immediately prior to the adoption of robotic assistance, during the surgeon’s fifth and sixth years of independent practice. For statistical comparison, a Kruskal-Wallis test was employed to assess differences in surgical time between the two robotic epochs and the conventional TKA group. Post hoc pairwise comparisons were conducted when appropriate. A significance level of 0.05 was used for all tests, and analyses were performed using Stata Version 17 (Released 2021; StataCorp LLC, College Station, TX, USA).

## Results

**Figure 2:**
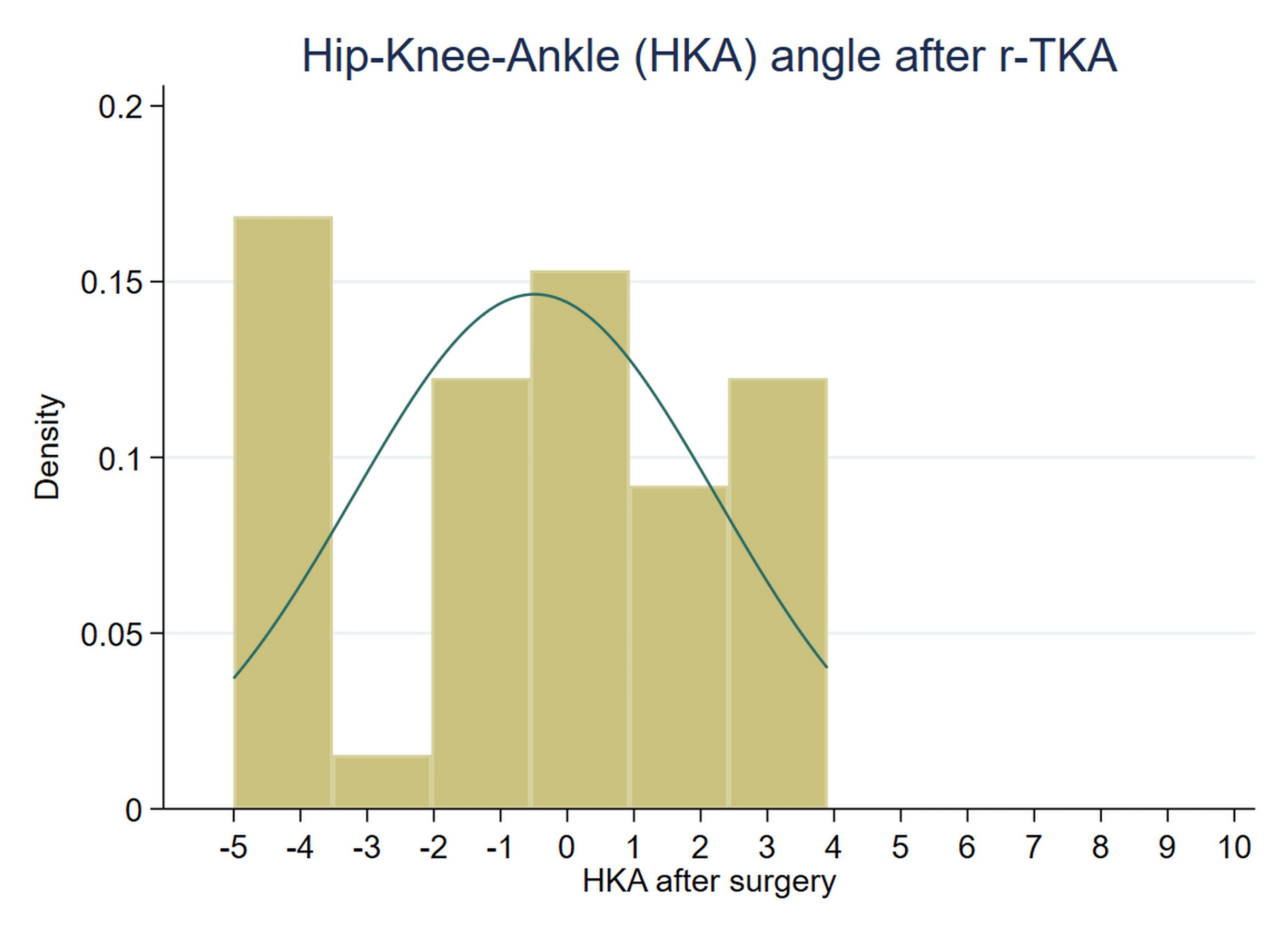
HKA angle after r-TKA Distribution of the final mechanical alignment (HKA angle) of the operated limb following r-TKA; Negative HKA values indicate valgus alignment, whereas positive values indicate varus alignment. HKA, hip-knee-ankle; r-TKA, robotic-assisted total knee arthroplasty

The first 50 patients who underwent r-TKA were included. The mean age was 67 years (standard deviation, 8.3; range, 52 to 82), and 32 patients (64%) were women. The median hospital stay was two days postoperatively (range, 2 to 3), and the postoperative HKA angle had a median of 0.5° of valgus (range, -5 to 4) (Figure 2).

Among the intraoperative events, one case required re-registration of anatomical landmarks due to incorrect positioning of the robotic guide, which was successfully resolved by repeating the registration process. In another case, a delay occurred due to the loosening of the screw that attaches the tracker to the Schanz pin during bone cutting with the saw; the Schanz pin itself remained stable. The issue was promptly resolved by re-tightening the screw. Both events occurred within the first 10 cases.

During follow-up, no thromboembolic events, periprosthetic joint infections, or hospital readmissions were observed. A single patient developed mild persistent serous drainage at the distal end of the wound, which was effectively managed with local wound care alone. Arthrocentesis revealed no signs of infection, and the patient maintained full range of motion without skin erythema or knee effusion. This minor complication was attributed to localized drainage along the Schanz pin tract.

**Figure 3:**
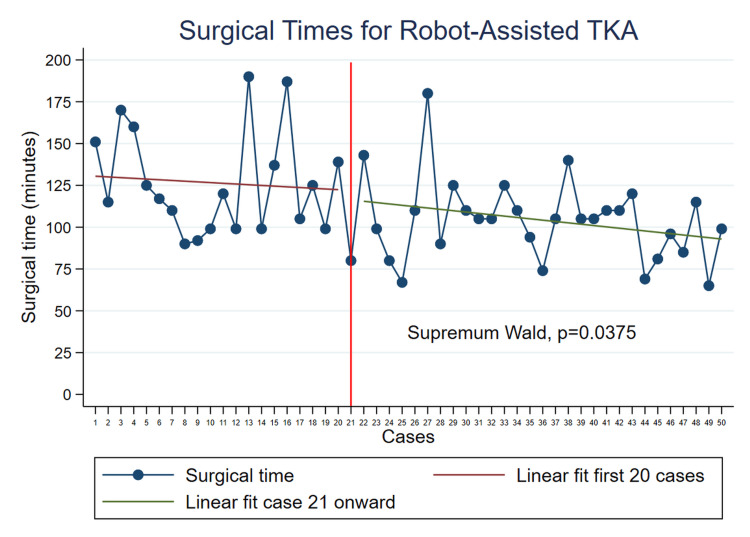
Surgical times for rTKA It displays the surgical time for each primary TKA case performed with robotic assistance, along with the linear fit for the two epochs identified in this study. Based on the Wald test, the significant change occurred at the 21st case. r-TKA, robotic-assisted total knee arthroplasty; TKA, total knee arthroplasty

Markov analysis identified two distinct phases, with a significant transition occurring at case 21. The first phase showed a median surgical time (Tcx) of 118 minutes (range, 90 to 190), whereas the second phase showed a median of 105 minutes (range, 65 to 180) (Figure 3).

**Figure 4:**
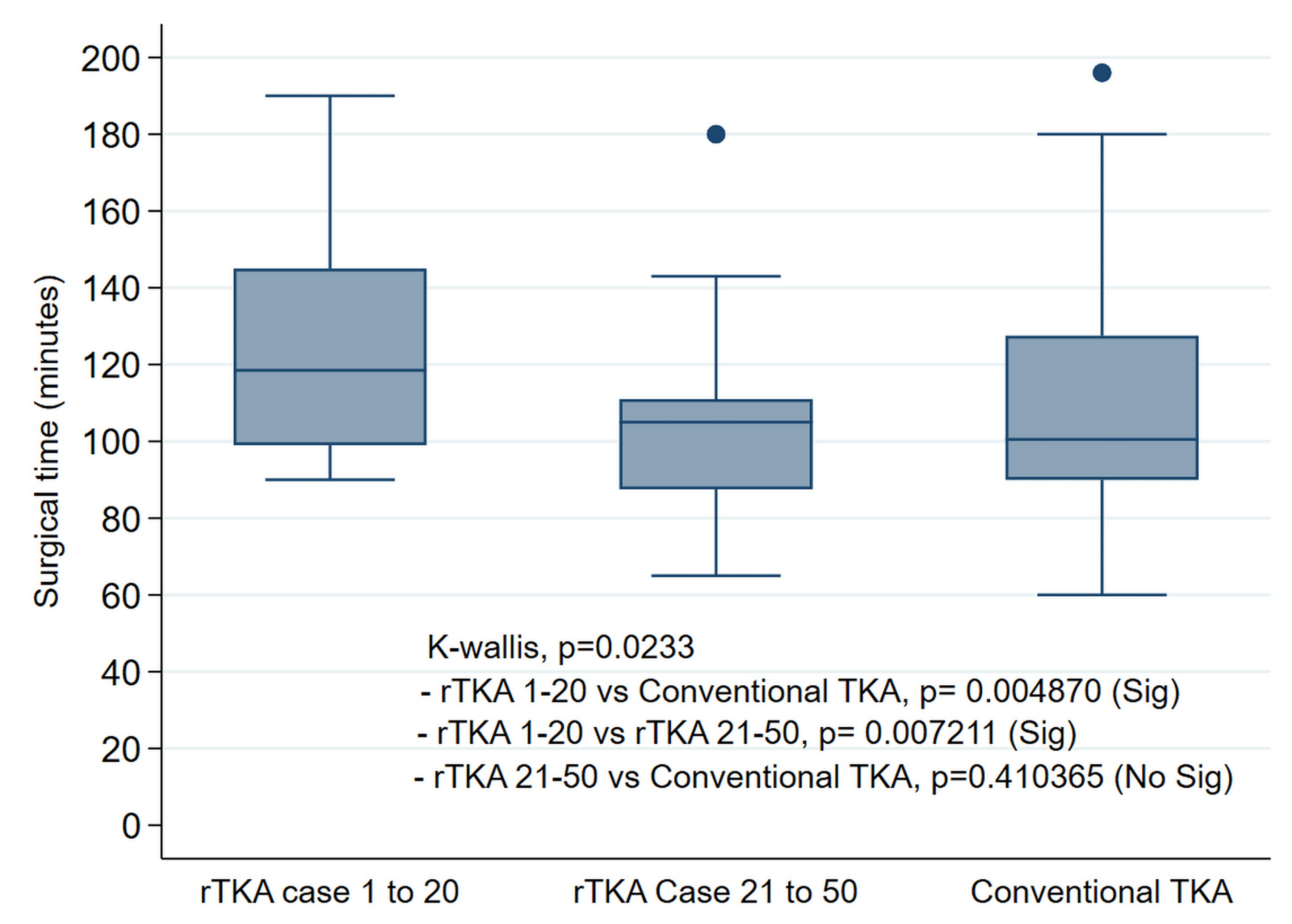
Surgical times Box plot showing the distribution of surgical time across the three cohorts included in the analysis: the first and second epochs of rTKA, and the conventional TKA cohort. No significant difference in surgical time was observed between the conventional cohort and the second epoch of rTKA. r-TKA, robotic-assisted total knee arthroplasty; TKA, total knee arthroplasty

Conventional TKA had a median time of 100 minutes (range, 60 to 196). The Kruskal-Wallis test revealed a significant difference among the three groups (p = 0.0233) (Figure 3). Post-hoc estimations indicated that the median surgical time during the first epoch of rTKA was significantly different from the median time during the second epoch of rTKA. However, no significant differences were found in the median surgical times between the second epoch of rTKA and conventional TKA (Figure 4).

## Discussion

The main finding of this study was the identification of a significant change in surgical time, which occurred after the 20th case. This finding indicates that, beyond this point, the surgical time for rTKA became comparable to that of conventional surgery. Additionally, no relevant medical complications related to the use of the robotic system were observed.

Surgical time is a crucial factor in TKA, as it directly impacts the efficiency of the operating room, resource allocation, and patient outcomes [17,18]. Robotic-assisted surgery has the potential to enhance implant alignment and minimize errors, thus improving overall surgical precision [7,8]. However, the perceived increase in operating time associated with robotic technology has been a major barrier to its widespread adoption [19-21].

In this study, the first epoch of rTKA cases (1-20) showed significantly longer surgical times compared to the second epoch (case 21 onward) and conventional TKA cases. These results suggest that, during the early stages of the learning curve, the time required to perform robotic-assisted surgery is longer, likely due to the surgeon's adaptation to the system and familiarity with the new technology. However, once the first 20 cases are completed, surgical time becomes comparable to conventional surgery, as demonstrated by a median of 100 minutes in the conventional cohort. This indicates that robotic technology does not result in a significant increase in operating time once experience is gained.

In the surgeon's experience, key factors for saving time include systematizing the process of reference point acquisition and the initial evaluation for the robot, achieving fluency in understanding point-by-point information and performing the robotic planning, and, finally, identifying critical details to enhance surgical flow, such as the orientation of the trackers, recognizing sources of error, and determining the optimal positioning for performing cuts guided by the robotic system. Additionally, it is essential to systematize checkpoints to ensure that the information provided by the robot aligns with the surgeon's perspective. This approach helps reduce the perception that the data is incorrect and, if necessary, allows for identifying where the error occurred, enabling the team to retrace their steps and proceed with a plan that aligns the robot's input with the surgeon's expertise.

Previous studies have reported learning curves ranging from 7 to 43 cases [12,22-24]. Mahure et al. [24] reported a learning curve of 10-20 cases for surgical time in a multicenter prospective study with 115 patients undergoing rTKA. They found a learning curve for surgical time, with no differences in complications, three-dimensional component position, or patient-reported outcome scores between the first and last group of patients. Vanlommel et al. [13] reported a learning curve of 6-11 cases to achieve surgical times comparable to standard rTKA, with similar 90-day complication rates and improved component positioning compared to manual TKA. They described the surgical results of three high-volume surgeons using the same robotic system as this study, so the results might be comparable. Interestingly, our findings suggest that a low-volume surgeon can reach a similar number of cases to achieve surgical time proficiency as reported in high-volume surgeon studies. This implies that surgeons' experience and understanding of the robotic system may be more influential than annual case volume in determining the learning curve.

Sodhi et al. [17] described the learning curve of two surgeons with a total of 240 rTKA cases, compared to a control group of 30 manual TKA cases done prior to initiating the study. The cases were sequentially grouped into 20 cases each, and the mean operative time was analyzed. They found significant differences in the first 20 rTKA cases compared to the manual TKA group for both surgeons. However, these differences were not found in the last 20 rTKA cases compared to the control group.

This study makes a contribution to the ongoing discussion by evaluating surgical time across a consecutive series of 50 rTKA cases performed by a single surgeon, focusing on how this parameter evolves along the learning curve. A key strength of this research is the use of advanced time-series analysis techniques, which appropriately account for the dependency between consecutive cases. Unlike traditional statistical methods, such as t-tests for comparing means, which assume data independence [14,25], time-series analysis recognizes that the surgical time for each case is intrinsically linked to the surgeon's experience gained from prior cases [15,26]. This dependency makes conventional inference techniques unsuitable for this type of analysis. To our knowledge, this is the first study to apply time-series methods to evaluate surgical time in this context. The use of Markov models provided a robust method to account for the sequential nature of surgical learning, offering insights that traditional mean-comparison methods cannot.

However, it is important to acknowledge that the surgical time data presented here are derived from a single surgeon with relatively low volume and less than a decade of experience in TKA, using one specific robotic system (ROSA^®^). It must be emphasized that, while caution is advised when generalizing these results, the findings are consistent with the range of learning curves reported in other studies. Notably, this study shows that a low-volume surgeon - performing approximately 30 cases per year - can achieve surgical time proficiency in rTKA within a similar number of cases as reported in studies involving high-volume surgeons. According to the 2023 American Joint Replacement Registry, the average annual volume for TKA procedures in the United States was 39 cases [27], which situates this study within a representative volume range. These findings suggest that annual surgical volume may not be the primary determinant of learning curve efficiency, and that factors such as overall surgical experience and familiarity with the robotic system may play a more significant role. This insight is particularly relevant for surgeons or institutions with volumes below the national average who are considering transitioning to rTKA.

## Conclusions

This study showed that surgical time in rTKA becomes comparable to conventional TKA after 20 cases, indicating that robotic assistance does not pose a long-term efficiency barrier. The initial increase in operative time reflects the learning curve, but once the surgeon adapts, procedural duration aligns with conventional techniques. By using time-series analysis, we captured the sequential nature of surgical learning more accurately than traditional methods. These findings help set realistic expectations for robotic adoption, emphasizing that, with proper training, robotic technology can be integrated without compromising efficiency.

Importantly, no major postoperative complications or hospital readmissions were observed in this early series. The intraoperative events related to the robotic system - such as the need to re-register anatomical landmarks or the loosening of the screw securing the tracker to the Schanz pin - were minor, occurred during the initial cases, and are easily correctable with improved familiarity and workflow optimization. These results support the feasibility and safety of implementing rTKA. These findings are particularly relevant for low- to moderate-volume surgeons, demonstrating that surgical efficiency with robotic assistance can be achieved within a case range similar to that reported for high-volume practices.
